# Supplementary material for: Covalent Adaptable Networks from Polyacrylates Based on Oxime–Urethane Bond Exchange Reaction
Source: Int J Mol Sci. 2024 Nov 30;25(23):12897. doi: 10.3390/ijms252312897 (PMC11641462; doi:10.3390/ijms252312897)
Supplement: Supplementary file 1 [file ijms-25-12897-s001.zip › ijms-3314370-supplementary.pdf]

Supplementary Materials

Covalent Adaptable Networks from Polyacrylates Based on Oxime-Urethane Bond Exchange Reaction

Yu Sotoyama, Naoto Iwata and Seiichi Furumi*

Department of Chemistry, Graduate School of Science, Tokyo University of Science,
1-3 Kagurazaka, Shinjuku, Tokyo 162-8601, Japan

*E-mail: furumi@rs.tus.ac.jp

Supplementary Figures and Tables

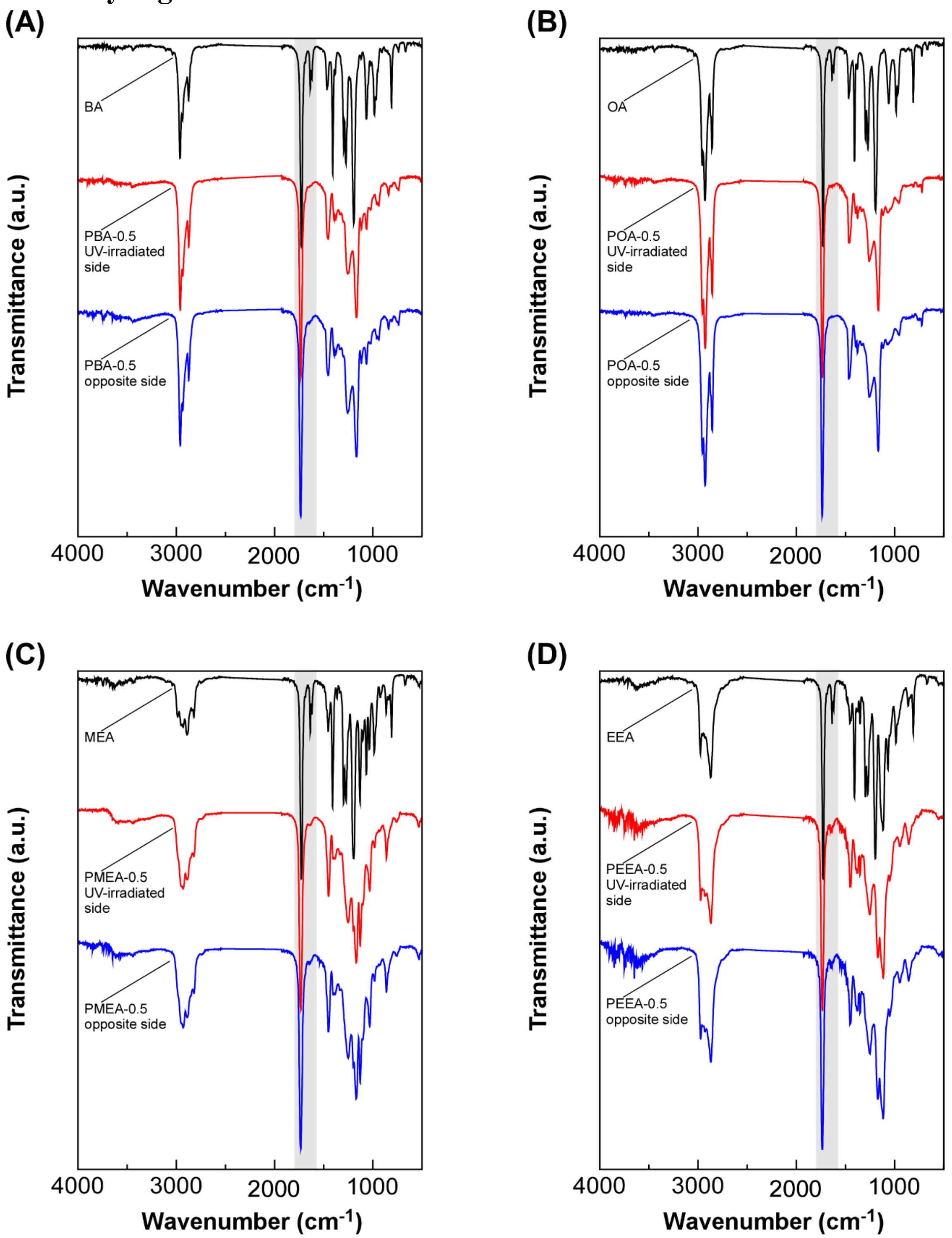

Figure S1. The representative attenuated total reflection Fourier transform infrared (ATR FT-IR) spectra of acrylate monomers and cross-linked films of PBA-0.5 (A), POA-0.5 (B), PMEA-0.5 (C), and PEEA-0.5 (D). Black lines denote the spectra of corresponding acrylate monomers. Red and blue lines denote spectra of cross-linked film of the UV-irradiate side and the opposite side, respectively.

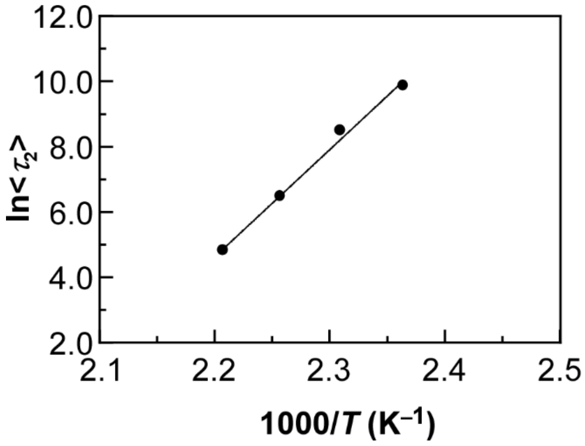

Figure S2. Arrhenius plot of $<\tau_2>$ of PBA-0.5, where solid line indicates the linear regression line of the plots to calculate the activation energy ($E_{a,2}$).

Table S1. Shift factors and fitting parameters of PBA-0.5.

T (ºC)	a_T	b_T	τ_1 (s)	τ_2 (s)	$<\tau_1>$ (s)	$<\tau_2>$ (s)	β_1	β_2	A_1	A_2
150	1.00	1.00	334	1.44×10^4	373	1.92×10^4	0.816	0.668	0.543	0.457
160	0.333	1.26	129	2.83×10^3	142	4.85×10^3	0.843	0.548	0.549	0.451
170	0.123	1.45	49.8	496	53.0	646	0.881	0.681	0.543	0.457
180	0.0565	1.96	18.2	118	19.0	123	0.914	0.924	0.523	0.477

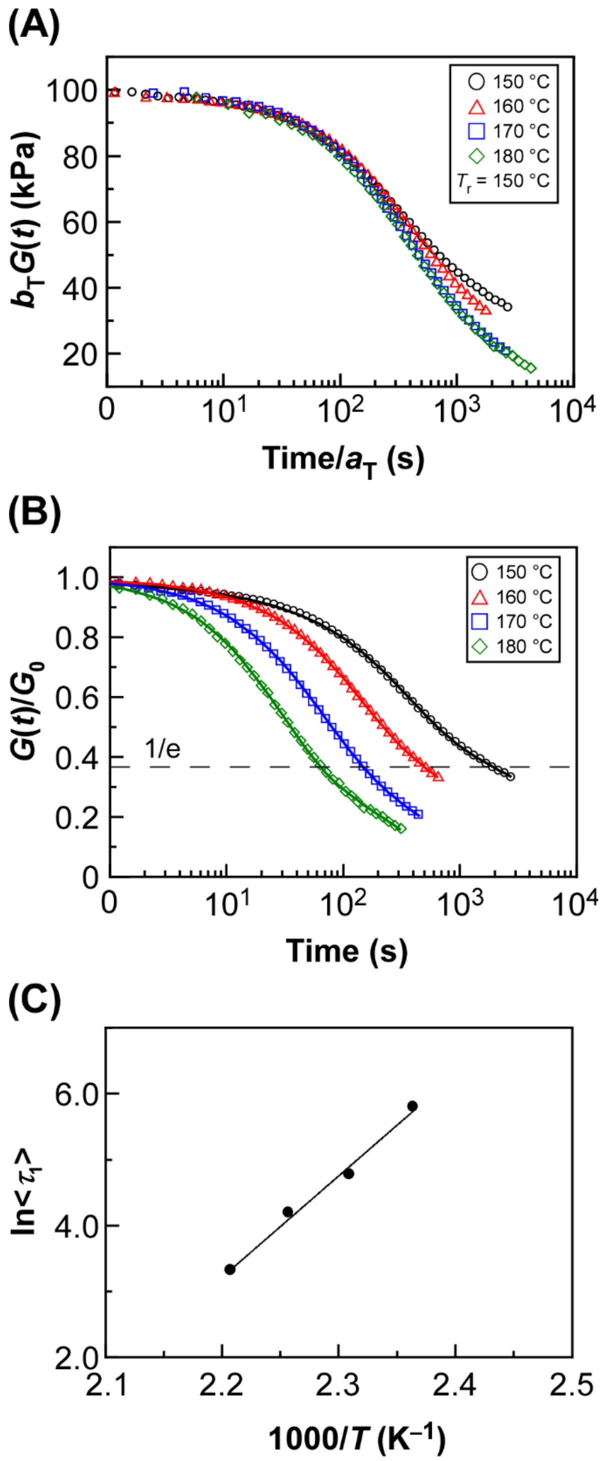

Figure S3. Results of stress relaxation measurements of PBA-1.0. (A) Master curve constructed at a reference temperature of 150 ºC. The values of a_T and b_T are horizontal and vertical shift factors, respectively. (B) Stress relaxation curves measured at the temperature between 150 ºC and 180 ºC. Solid lines represent the fitting curves based on Equation 1. (C) Arrhenius plot of <τ_1>. Solid line represents the linear regression line of the plots.

Table S2. Shift factors and fitting parameters of PBA-1.0.

T (ºC)	a_T	b_T	τ_1 (s)	τ_2 (s)	$<\tau_1>$ (s)	$<\tau_2>$ (s)	β_1	β_2	A_1	A_2
150	1.00	1.00	329	6.67×10^3	329	2.31×10^4	1.00	0.394	0.323	0.677
160	0.367	1.02	118	1.41×10^3	117	2.25×10^3	1.00	0.576	0.374	0.626
170	0.168	1.17	61.2	45.8	66.2	46.0	0.858	0.993	0.651	0.349
180	0.0727	1.96	26.8	354	27.5	390	0.941	0.833	0.612	0.388

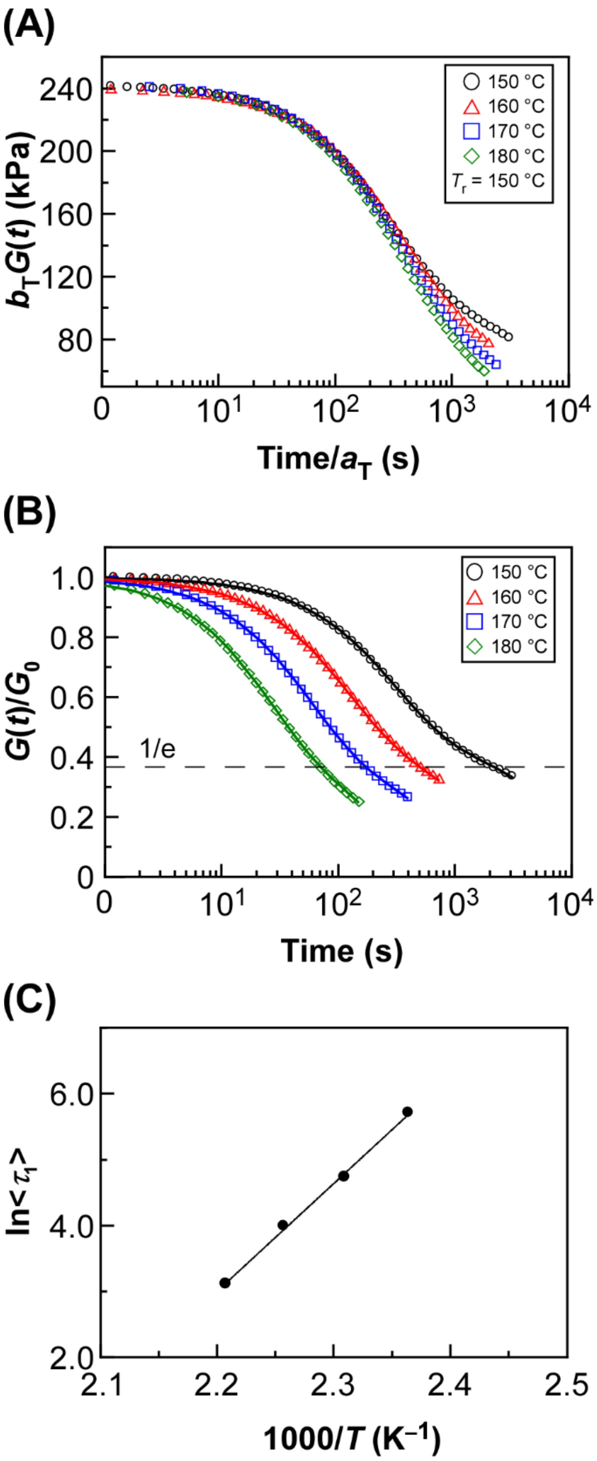

Figure S4. Results of stress relaxation measurements of PBA-2.0. (A) Master curve constructed at a reference temperature of 150 ºC. The values of a_T and b_T are horizontal and vertical shift factors, respectively. (B) Stress relaxation curves measured at the temperature between 150 ºC and 180 ºC. Solid lines represent the fitting curves based on Equation 1. (C) Arrhenius plot of <τ_1>. Solid line represents the linear regression line of the plots.

Table S3. Shift factors and fitting parameters of PBA-2.0.

T (ºC)	a_T	b_T	τ_1 (s)	τ_2 (s)	$<\tau_1>$ (s)	$<\tau_2>$ (s)	β_1	β_2	A_1	A_2
150	1.00	1.00	290	1.02×10^4	301	1.16×10^4	0.921	0.800	0.511	0.489
160	0.355	1.04	110	1.99×10^3	114	2.13×10^3	0.935	0.869	0.514	0.486
170	0.164	1.13	52.6	829	54.0	878	0.943	0.890	0.561	0.439
180	0.0798	1.21	22.5	224	22.5	245	0.998	0.845	0.495	0.505

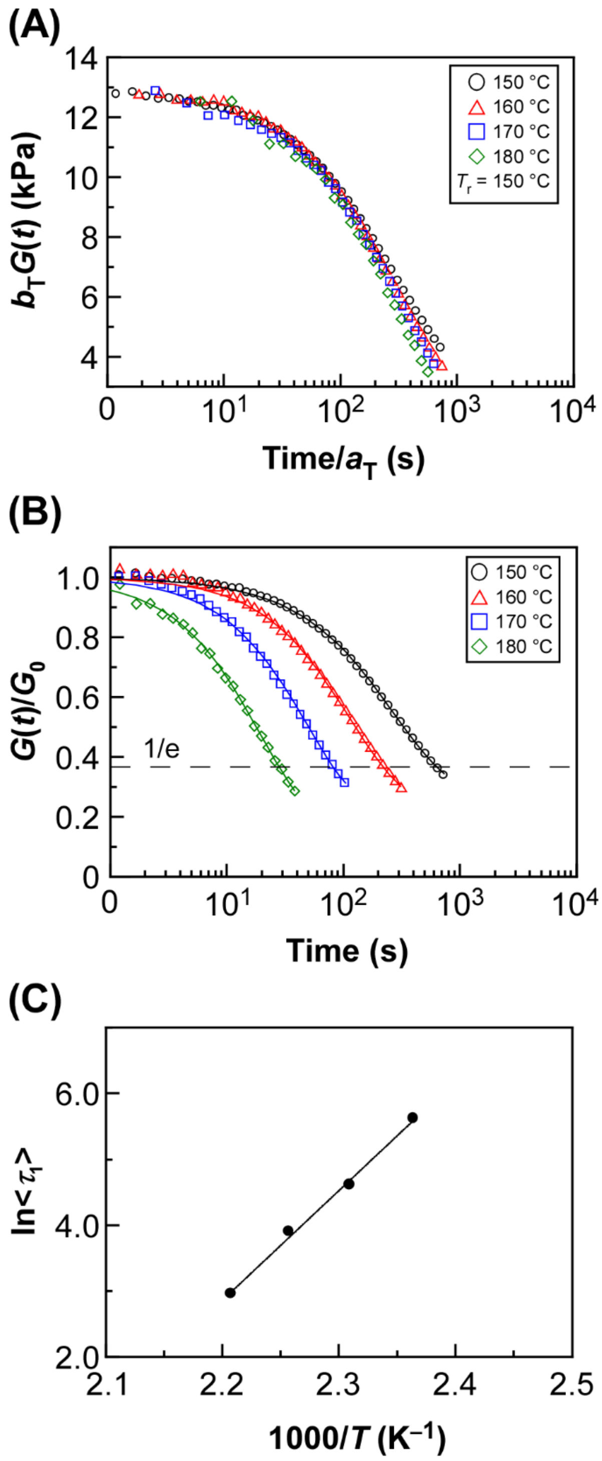

Figure S5. Results of stress relaxation measurements of POA-0.5. (A) Master curve constructed at a reference temperature of 150 ºC. The values of a_T and b_T are horizontal and vertical shift factors, respectively. (B) Stress relaxation curves measured at the temperature between 150 ºC and 180 ºC. Solid lines represent the fitting curves based on Equation 1. (C) Arrhenius plot of $<\tau_1>$. Solid line represents the linear regression line of the plots.

Table S4. Shift factors and fitting parameters of POA-0.5.

T (ºC)	a_T	b_T	τ_1 (s)	τ_2 (s)	$<\tau_1>$ (s)	$<\tau_2>$ (s)	β_1	β_2	A_1	A_2
150	1.00	1.00	260	1.39×10^4	260	1.51×10^4	0.898	0.849	0.685	0.315
160	0.424	1.22	101	1.26×10^3	101	1.26×10^3	1.00	1.00	0.650	0.350
170	0.161	1.55	49.5	1.19×10^3	49.5	1.19×10^3	1.00	1.00	0.780	0.220
180	0.0679	2.35	19.2	815	19.2	815	1.00	1.00	0.816	0.184

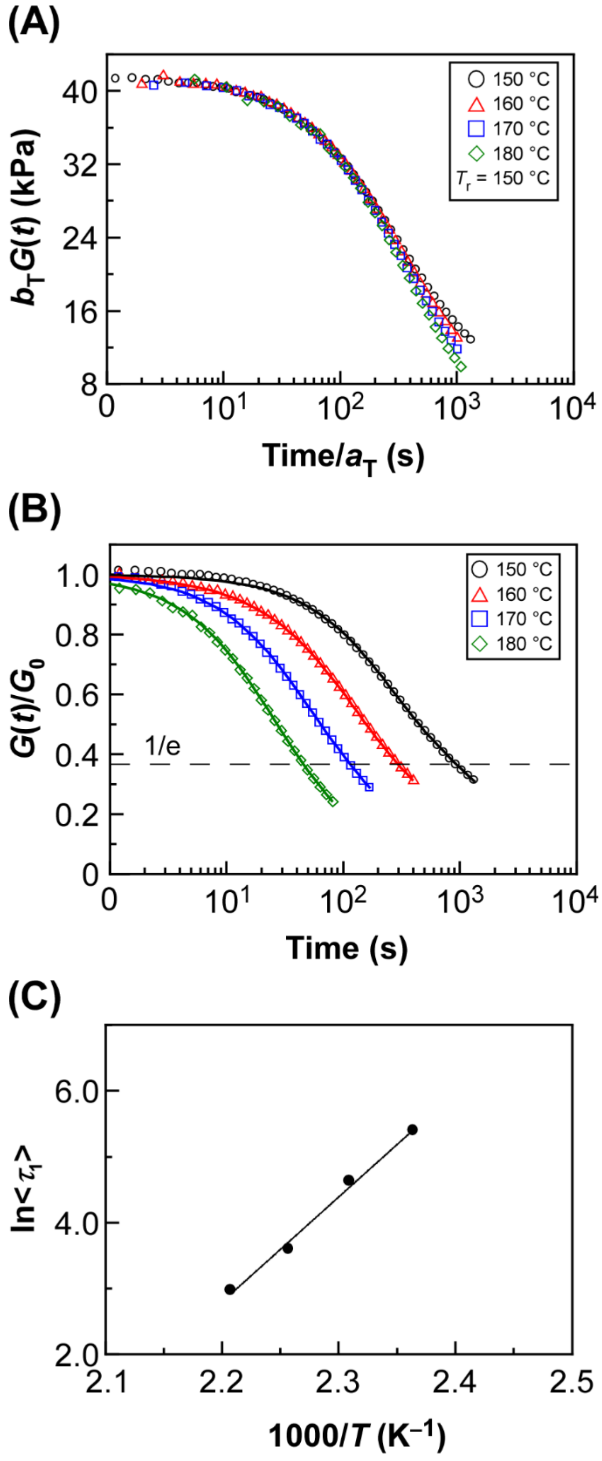

Figure S6. Results of stress relaxation measurements of POA-1.0. (A) Master curve constructed at a reference temperature of 150 ºC. The values of a_T and b_T are horizontal and vertical shift factors, respectively. (B) Stress relaxation curves measured at the temperature between 150 ºC and 180 ºC. Solid lines represent the fitting curves based on Equation 1. (C) Arrhenius plot of <τ_1>. Solid line represents the linear regression line of the plots.

Table S5. Shift factors and fitting parameters of POA-1.0.

T (ºC)	a_T	b_T	τ_1 (s)	τ_2 (s)	<τ_1> (s)	<τ_2> (s)	β_1	β_2	A_1	A_2
150	1.00	1.00	220	2.52×10^3	220	2.52×10^3	1.00	1.00	0.477	0.523
160	0.397	1.18	97.7	933	102	949	0.913	0.962	0.536	0.464
170	0.165	1.40	36.3	265	36.3	265	1.00	1.00	0.463	0.537
180	0.0753	1.70	19.5	122	19.5	122	1.00	1.00	0.543	0.547

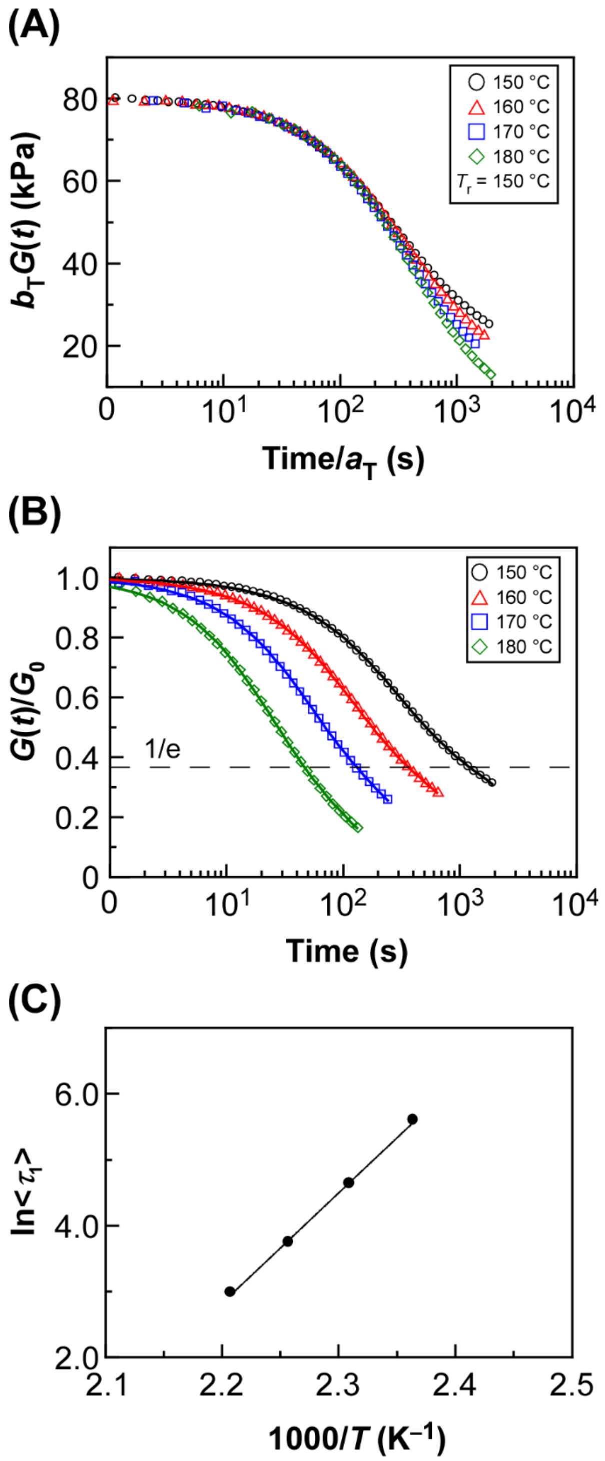

Figure S7. Results of stress relaxation measurements of POA-2.0. (A) Master curve constructed at a reference temperature of 150 ºC. The values of a_T and b_T are horizontal and vertical shift factors, respectively. (B) Stress relaxation curves measured at the temperature between 150 ºC and 180 ºC. Solid lines represent the fitting curves based on Equation 1. (C) Arrhenius plot of <τ_1>. Solid line represents the linear regression line of the plots.

Table S6. Shift factors and fitting parameters of POA-2.0.

T (ºC)	a_T	b_T	τ_1 (s)	τ_2 (s)	$<\tau_1>$ (s)	$<\tau_2>$ (s)	β_1	β_2	A_1	A_2
150	1.00	1.00	253	4.69×10^3	270	5.11×10^3	0.881	0.849	0.508	0.492
160	0.378	1.10	99.6	1.23×10^3	103	1.28×10^3	0.932	0.913	0.516	0.484
170	0.167	1.22	41.9	367	42.3	372	0.979	0.970	0.505	0.495
180	0.0683	1.47	19.8	123	19.8	127	0.999	0.945	0.528	0.472

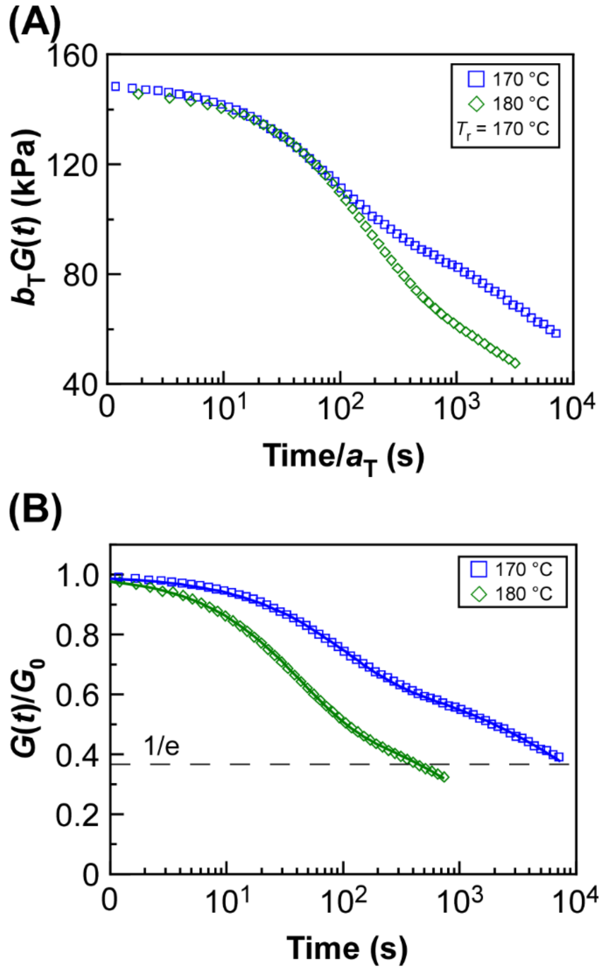

Figure S8. Results of stress relaxation measurements of PMEA-0.5. Stress relaxation measurements were only performed at two temperatures due to extremely long relaxation times. Therefore, the Arrhenius plot could not be constructed. (A) Master curve constructed at a reference temperature of 170 ºC. The values of a_T and b_T are horizontal and vertical shift factors, respectively. (B) Stress-relaxation curves at 170 °C or 180 °C. Solid lines represent the fitting curves calculated using Equation 1.

Table S7. Shift factors and fitting parameters of PMEA-0.5.

T (ºC)	a_T	b_T	τ_1 (s)	τ_2 (s)	$\langle\tau_1\rangle$ (s)	$\langle\tau_2\rangle$ (s)	β_1	β_2	A_1	A_2
170	1.00	1.00	92.7	2.03×10^4	99.6	4.71×10^4	0.869	0.464	0.296	0.704
180	0.231	1.40	39.2	2.11×10^3	42.1	2.79×10^3	0.867	0.670	0.477	0.523

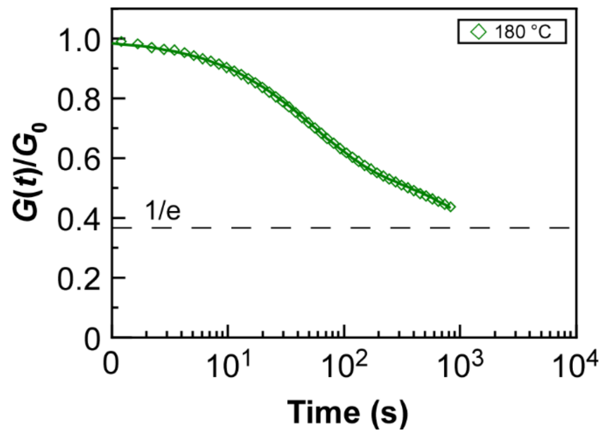

Figure S9. Stress relaxation curve of PEEA-0.5 at 180 °C. Stress relaxation test was only performed at one temperature due to extremely long relaxation time. Therefore, the Arrhenius plot could not be constructed. Solid line represents the fitting curve calculated using Equation 1.

Table S8. Fitting parameters of PEEA-0.5.

T (ºC)	τ_1 (s)	τ_2 (s)	$<\tau_1>$ (s)	$<\tau_2>$ (s)	β_1	β_2	A_1	A_2
180	48.5	4.13×10^3	52.1	5.46×10^3	0.867	0.669	0.390	0.610

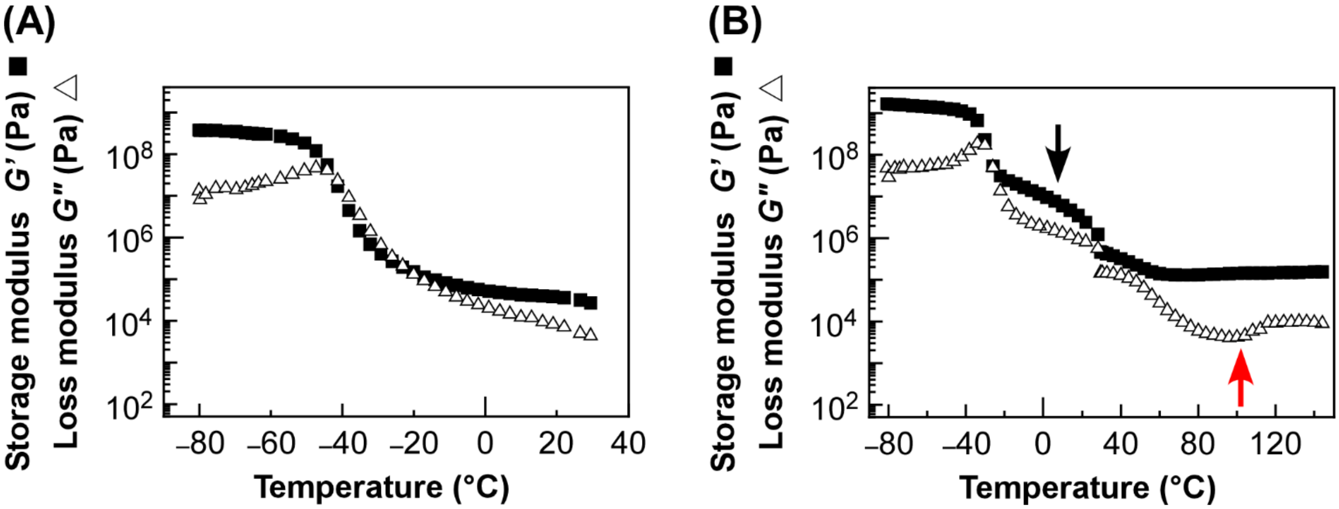

Figure S10. Representative temperature dependence of the storage (*G*') and loss (*G*'') moduli of PBA-0.5 (A), and PMEA-0.5 (B). In PMEA-0.5, peaks were observed for both *G*' and *G*'' at 0–40 ºC (black arrow) and for *G*'' at 80–120 ºC (red arrow).

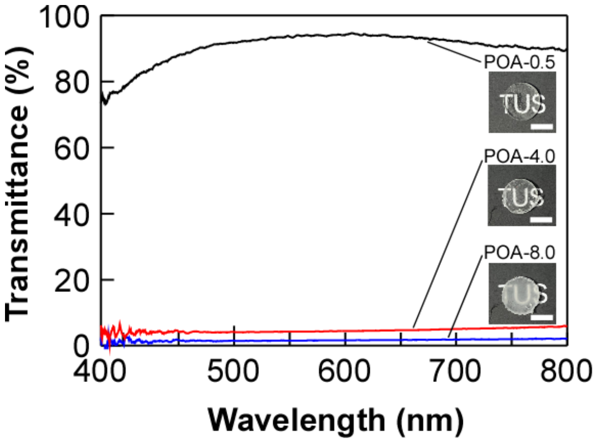

Figure S11. Transmission spectra of POA-0.5 (black line), POA-4.0 (red line), and POA-8.0 (blue line). Insets are the photographs of POA-0.5, POA-4.0, and POA-8.0. White scale bars denote 5mm.

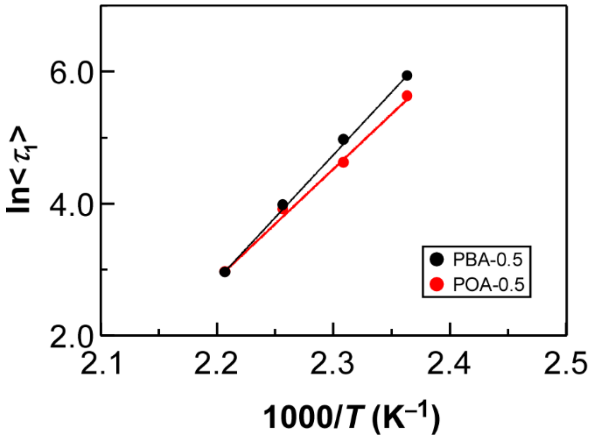

Figure S12. Comparison of Arrhenius plots of <τ_1> for PBA-0.5 (black circles) and POA-0.5 (red circles). Solid lines represent the linear regression lines of the corresponding plots.

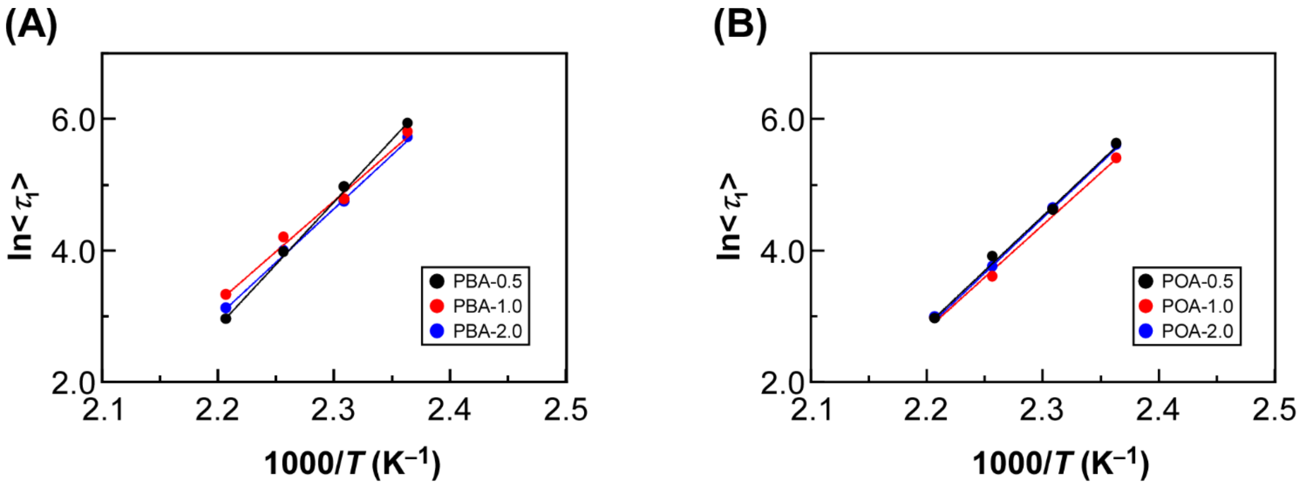

Figure S13. (A) Comparison of Arrhenius plots of <τ_1> for PBA-0.5 (black circles) and PBA-1.0 (red circles), and PBA-2.0 (blue circles). Solid lines represent the linear regression lines of the corresponding plots. (B) Comparison of Arrhenius plots of <τ_1> for POA-0.5 (black circles) and POA-1.0 (red circles), and POA-2.0 (blue circles). Solid lines represent the linear regression lines of the corresponding plots.

Table S9. Results of compression test of POA-0.5, POA-1.0, and POA-2.0.

Sample code	Young's modulus (kPa)	Hysteresis loss rate (%)
POA-0.5	12.4	22.8
POA-1.0	15.6	23.3
POA-2.0	13.8	25.4

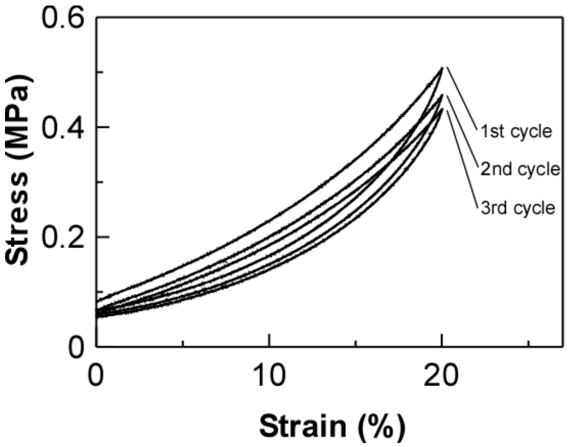

Figure 14. Stress-strain curves of POA-0.5 in repeated cycles as compressing the strain up to 20%

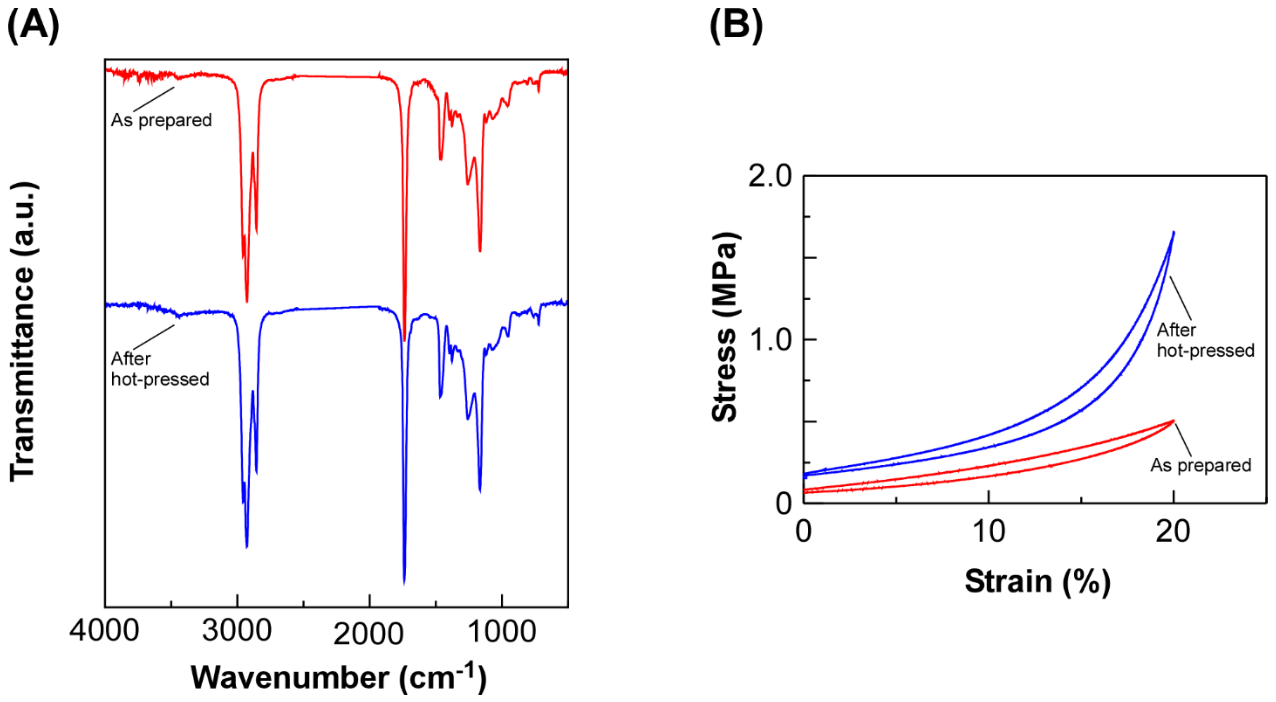

Figure S15. Results of reprocessing test of POA-0.5. (A) ATR FT-IR spectra measured for as-prepared sample (red line) and hot-pressed sample (blue line). (B) Stress-strain curves measured of as-prepared sample (red line) and hot-pressed sample (blue line) as compressing the strain up to 20%.

Table S10. Fitting parameters of POA-0.5 after reprocessing test.

T (ºC)	τ_1 (s)	τ_2 (s)	$<\tau_1>$ (s)	$<\tau_2>$ (s)	β_1	β_2	A_1	A_2
150	252	3.79×10^4	252	3.78×10^3	1.00	1.00	0.460	0.540
160	129	3.74×10^3	130	4.15×10^3	0.976	0.868	0.600	0.400
170	67.5	1.04×10^3	78.9	1.44×10^4	0.767	0.642	0.802	0.198
180	18.7	120	21.8	120	1.00	1.00	0.611	0.389

Table S11. Rheological property and activation energies of POA-0.5 after reprocessing test.

G' (kPa)[1]	$E_{a,1}$ (kJ/mol)[2]	$E_{a,2}$ (kJ/mol)[3]
17.8	125	141

[1] Storage modulus (G') at 1Hz measured at 30 ºC. [2] Activation energy ($E_{a,1}$) of bond exchange. [3] Activation energy ($E_{a,2}$) of relaxation arising from melting of aggregated DOUDA.
